# Tricarboxylate Citrate Transporter of an Oleaginous Fungus *Mucor circinelloides* WJ11: From Function to Structure and Role in Lipid Production

**DOI:** 10.3389/fnut.2021.802231

**Published:** 2021-12-09

**Authors:** Wu Yang, Aabid Manzoor Shah, Shiqi Dong, Caili Sun, Huaiyuan Zhang, Hassan Mohamed, Xiuzhen Gao, Huirong Fan, Yuanda Song

**Affiliations:** ^1^Colin Ratledge Center for Microbial Lipids, School of Agriculture Engineering and Food Sciences, Shandong University of Technology, Zibo, China; ^2^Tianjin Key Laboratory of Radiation Medicine and Molecular Nuclear Medicine, Institute of Radiation Medicine, Chinese Academy of Medical Sciences and Peking Union Medical College, Tianjin, China; ^3^Department of Botany and Microbiology, Faculty of Science, Al-Azhar University, Assiut, Egypt

**Keywords:** *Mucor circinelloides*, tricarboxylate citrate transporter, molecular dynamics, citrate efflux, molecular modeling

## Abstract

The citrate transporter protein (CTP) plays an important role in citrate efflux from the mitochondrial matrix to cytosol that has great importance in oleaginous fungi. The cytoplasmic citrate produced after citrate efflux serves as the primary carbon source for the triacylglycerol and cholesterol biosynthetic pathways. Because of the CTP's importance, our laboratory has extensively studied its structure/function relationships in *Mucor circinelloides* to comprehend its molecular mechanism. In the present study, the tricarboxylate citrate transporter (Tct) of *M. circinelloides* WJ11 has been cloned, overexpressed, purified, kinetically, and structurally characterized. The Tct protein of WJ11 was expressed in *Escherichia coli*, isolated, and functionally reconstituted in a liposomal system for kinetic studies. Our results showed that Tct has a high affinity for citrate with Km 0.018 mM. Furthermore, the *tct* overexpression and knockout plasmids were created and transformed into *M. circinelloides* WJ11. The mitochondria of the *tct*-overexpressing transformant of *M. circinelloides* WJ11 showed a 49% increase in citrate efflux, whereas the mitochondria of the *tct*-knockout transformant showed a 39% decrease in citrate efflux compared to the mitochondria of wild-type WJ11. To elucidate the structure-function relationship of this biologically important transporter a 3D model of the mitochondrial Tct protein was constructed using homology modeling. The overall structure of the protein is V-shaped and its 3D structure is dimeric. The transport stability of the structure was also assessed by molecular dynamics simulation studies. The activity domain was identified to form hydrogen bond and stacking interaction with citrate and malate upon docking. Tricarboxylate citrate transporter has shown high binding energy of −4.87 kcal/mol to citric acid, while −3.80 kcal/mol to malic acid. This is the first report of unraveling the structural characteristics of WJ11 mitochondrial Tct protein and understanding the approach of the transporting toward its substrate. In conclusion, the present findings support our efforts to combine functional and structural data to better understand the Tct of *M. circinelloides* at the molecular level and its role in lipid accumulation.

## Introduction

Citrate, an intermediate of Krebs cycle, is a precursor to lipid and cholesterol biosynthesis as well as a nexus point between glucose and lipid pathways (1–4). In oleaginous fungi, extra citrate in the mitochondria had to be transported into the cytosol and metabolized by ATP-citrate lyase to form acetyl-CoA, an important precursor for lipid biosynthesis (5, 6). Intracellular citrate influences the activity of key enzymes in fatty acid (FA) oxidation and glycolysis (7). Citrate products, such as malonyl CoA, play important signaling roles in energy expenditure regulation (8). Citrate cannot diffuse through the mitochondrial membrane therefore transport process is facilitated by mitochondrial citrate transport system (7). The ability of transporters to bind to their specific substrate is a major mechanism for modifying transport activity (9). To better understand the citrate binding potential to the tricarboxylate citrate transporter (Tct) in oleaginous *Mucor circinelloides*, its Tct binding properties must be known. *M. circinelloides* is a dimorphic Zygomycete fungus, a model organism for lipid studies that has been researched for the last 35 years (10, 11). Its biotechnological interest as a major source of carotenes and lipids especially γ-linolenic acid has gained the interest of researchers all over the world (6).

In the Zygomycota phylum, *M. circinelloides* has the most diverse repertoire of molecular tools (12, 13). This includes self-replicating plasmid-mediated genetic transformation, *Agrobacterium*-mediated integrative transformation, the generation of knockout mutants, and the use of RNAi-based procedures to suppress gene function (12). The known *Mucor* genome sequence aids in the identification and study of genes and proteins involved in the aforementioned processes, as well as the production of lipids useful in the production of biodiesel (13).

For decades, computational methods for predicting protein structure and ligand-protein interactions have been used successfully in biochemical research. Five transporters involved in mitochondrial citrate transportation were discovered in *M. circinelloides* based on their predicted function in the TCDB. These included a citrate transporter (Ct), also known as CiC, a tricarboxylate carrier (Tct), that was found transporting citrate out of mitochondria, and a malate transporter (Mt), which may be involved in malate translocation (14, 15). Recently, it was reported that the overexpressing of Tct in *M. circinelloides* resulted in a 68% increase in lipid production (16), implying that Tct facilitated citrate transport from mitochondria in *M. circinelloides*, which was associated with high lipid biosynthesis. Tricarboxylate carrier protein of *M. circinelloides* when blasted, was found as Tct (Mtc family) belongs to the mitochondrial carrier large family (MC) that have a specific character contains three times tandemly repeated 100 residue domain, with two hydrophobic segments and a signature sequence motif PX [D/E]XX [K/R]X [K/R] (20–30 residues) [D/E]GXXXX [W/Y/F][K/R]G (PFAM03820) (17, 18).

In this study, we examined the genome of *M. circinelloides* WJ11 a high lipid-producing strain to identify homology modeling, molecular docking, molecular dynamics, and citrate efflux of tricarboxylate carrier (Tct) involved in the citrate transport system. In order to gain a better understanding of the functions of the Tct transporter in the mitochondrial citrate transport system, general properties such as protein sequence identity and domain structure were studied *in silico* as well as their expression profiling *in vitro*. Our study confirmed that Tct contributes to the efflux of citrate from mitochondria that provide enough carbon sources for cell utilization thus have a significant impact on lipid accumulation

## Materials and Methods

### Strains, Media, and Culture Conditions

Competent *Escherichia coli* BL21 (DE3) cells were used for *tct* gene heterologous expression (19). For fungal transformation experiments, *M. circinelloides* WJ11 (CCTCC No. M2014424; China Center for Type Culture Collection) was used as the recipient strain for *tct* gene overexpression and knockout.

*Mucor circinelloides* cultures were inoculated at approximately 10^6^-10^7^ spores/ml into 150 ml K&R seed medium (1 L flask with baffles) and incubated at 28°C for 24 h in an incubator shaker with 150 rpm (revolution/min). The 10% seed culture was then inoculated into a 1.5 L modified K&R fermentation medium in a 2 L bioreactor (BioFlo/CelliGen115, New Brunswick Scientific, Edison, NJ, USA) (20). At 72 h, culture samples of transformed strain were taken for further experimentation (5, 15, 21).

### Transport Activity Determination of Tct Reconstituted Liposomes

#### Heterologous Expression and Purification

The entire *tct* gene sequence was optimized (according to *E. coli* codon usage), synthesized, and subcloned into the expression vector pET30a(+). The plasmid pET30a(+)-*tct* was made using the following cloning strategy: pET30a-NdeI-ATG-ct-Histag-Stop codon-HindIII-pET30a.The heat-shock method was used for transformation in the BL21 (DE3) strain. The pET30a-*tct* recombinant strain BL21 (DE3) was inoculated into LB broth medium supplemented with kanamycin incubated at 37°C for 16 h (22). Pre-inoculum (1 ml) was inoculated into 100 ml LB_kan_ and culture was grown at 37 °C until OD600 was reached 1.2. The expression of pET30a-tct were induced by IPTG at 15°C for 16 h before being harvested by centrifugation (5,000 g for 10 min). Cell pellets were resuspended in lysis buffer (50 mM Tris, 150 mM NaCl, 5% glycerol, pH 8.0), then sonicated for 10 min. Urea was then used to dissolve the precipitate. Denatured protein was obtained in a single step using a Ni-column purification method (23). The target protein was renatured and sterilized by passing it through a 0.22 μm filter. Isolated pure protein was dissolved in 1X PBS (Phosphate-buffered saline), pH 7.4, 10% glycerol, and 0.5 M L-arginine. The concentration was determined using the Bradford protein assay, which used BSA (Bovine serum albumin) as the standard. SDS-PAGE and Western blot (GenScript, Cat. No. A00186) were used to analyze samples of whole cell lysate, supernatant, and debris. Standard SDS-PAGE and western blot confirmation were used to determine protein purity and molecular weight (Supplementary Figure 1).

#### Tct Liposome Reconstitution and Transport Assay

The liposomes were prepared by adding 232, 58, and 94 mg of soybean lecithin, cholesterol, Tween 80, respectively, in 15 ml mixture of chloroform: methanol (3:1). This mixture was dried by rotavapor at 50°C followed by the addition of 20 ml of 20 mM phosphate buffer. The resulting solution was placed in the ultrasonic bath for 10 min.

The solubilized recombinant protein was reconstituted into liposomes after being diluted three times with a buffer containing 3% Triton X-114 (w/v), 20 mM Na_2_SO_4_, and 10 mM piperazine-1,4-bisethanesulfonic acid (PIPES, pH 7.0). The liposome system was designed as follows: 1% TritonX-114, ultrasound-prefabricated liposome, 20 mM PIPES, 0.8 mg cardiolipin, and water replenishment to a final volume of 700 μl. These components were gently mixed, and the mixture was recycled 13 times before being passed through a hydrophobic chromatography column (Bio-Rad Beads SM-2). The substrates and 10 mM PIPES (pH 7.0) were used to pre-equilibrate the columns (24–26). The substrate in this case is to be embedded in a liposome that exchanges citrate. Except for the passages through the column, which were performed at room temperature, all other operations were carried out at 4°C. The amount of purified protein reconstructed into the liposome was determined using the method described by Vito et al. (27), and 11.2% of the protein was added to the reconstructed mixture. Immediately before transport, a given proteoliposomal sample was thawed, sonicated on ice, and passed through Sephadex G-75 columns pre-equilibrated with buffer (10 mM PIPES and 50 mM NaCl pH 7.0) to remove the external citrate and other substrates.

To begin transport at 25°C, radioactive [^14^C] citrate (PerkinElmer Life Sciences) was added to either substrate-loaded (exchange) or empty proteoliposomes. The reaction was stopped by adding 20 mM pyridoxal 5'-phosphate, which completely and rapidly inhibits the activity of several MCTs (28, 29). The inhibitor was added together with the [^14^C] citrate at the start of the controls using the “inhibitor-stop” method (24). Finally, Sephadex G-75 was used to remove the external radioactivity from the protein liposomes, and the radioactivity of the protein liposomes was measured using a Liquid Scintillation Analyzer (PerkinElmer, Tri-carb 4910TR) (26, 30). The linear regression analysis of the transport results yielded the Km-values.

### Mitochondrial Transport Properties of Tct Mutants in WJ11

#### Construction of Over-Expression and Knockout Recombinant Mutants

The previous research work of our lab designed plasmids pMAT2085 and pMAT2060 for *tct* gene over-expression and knockout, respectively (unpublished data). The strain MU65, a uridine auxotrophic strain derived from WJ11 were transformed with pMAT2085 and pMAT2060 (31). To create the knockout strain, a plasmid carrying a selectable marker (pyrF) flanked by 1 kb of the tct gene's up and downstream regions was constructed. The selectable marker pyrF was amplified from M. circinelloides WJ11 strain. The three fragments (UP stream of tct, Downstream of tct and pyrF) were joined by overlap extension polymerase and the resultant fragment was cloned in pMAT2060. Restriction fragments from plasmid containing the pyrF gene, used as a selective marker, flanked by 1 kb sequences of the adjacent regions of the tct gene to allow homologous recombination were used to transform the MU65 strain, which is auxotrophic for uracil. After many vegetative cycles in the selective medium, transformed strains were selected and validated by PCR.

#### Mitochondrial Isolation

Transformed *M. circinelloides* were grown for 72 h in fermenter and the cell biomass was filtered, washed, and the mitochondria were isolated by the method as discussed in our previous research (17).

#### Viability Test for Isolated Mitochondria

To detect the mitochondrial viability, NADH and fumarate were added to the mitochondria suspension [100 μl NADH (0.5 mmol), 20 μl fumarate (7 mmol), 70 μl 1 × PBS buffer (pH 7.4), and 10 μl mitochondria]. After 20 min of incubation, the absorbance of the biochemical reaction mixture was measured with a microporous plate absorbance spectrophotometer (Bio-Rad xMark^TM^) at 340 nm using the enzymatic kinetic method (21).

#### Measuring Mitochondrial Transport Activity

The mitochondrial suspension was pre-incubated in a 30°C water bath for 3 min to “load” the mitochondria with [^14^C] citrate before adding the substrate (21). The reaction is started by adding malate or α-ketoglutarate at the same time, and it is stopped by rapid centrifugation. The incorporation of [^14^C] citrate radiolabel into mitochondrial pellets and the disappearance of [^14^C] citrate radiolabel from incubations were used to determine citrate uptake. To achieve a final volume of 1.0 ml, 10 mM substrates were added simultaneously. After 5 min, the reactions were stopped by rapidly separating the mitochondria from the incubation mixture using the same centrifugation conditions.

### Sequence and Structure Analysis of tct Gene

Tricarboxylate citrate transporter amino acid sequences were obtained from Uniprot and physicochemically predicted using ProtParam (http://web.expasy.org/protparam/), while hydrophobicity was predicted using ProtScale (http://web.expasy.org/protscale/). Gene annotations were used to identify putative mitochondrial transporter genes in *M. circinelloides* WJ11 using databases such as the Kyoto Encyclopedia of Genes and Genomes (KEGG), the National Center for Biotechnology Information (NCBI), non-redundant proteins (NR), protein families (Pfam), and the transporter classification database (TCDB). The amino acid sequences of Cts of yeasts that resemble *Mucor* and mitochondrial transporter family members whose crystal structures have been determined were aligned with our sequenced Tct using the NCBI-PubMed database search results. Pairwise Sequence Alignment was used to analyze the homology of the sequences, which were then aligned with ClustalW and ESPript.

### Homology Modeling and Model Quality Evaluation

LOMETS searched the PDB database (www.pdb.org) for proteins with similar topology to the target protein sequences that had already been resolved experimentally (32). The protein structures with a high Z-Score were chosen from the search results as the template structures for subsequent modeling calculations, and the results are shown in Supplementary Table 1.

The Tct protein structure was predicted by the protein topological similarity principle using the obtained template protein 3D structure files and sequence comparison files. Tricarboxylate citrate transporter protein structures were predicted separately using the I-TASSER threading method to obtain the target proteins' 3D structures. During the prediction calculation, five different target protein structures were generated, and each target protein structure was evaluated for plausibility using the C-score, target protein model with the highest score being chosen for further calculations.

Gromacs 4.6 kinetics software was used to optimize the target protein structure, either for the amino acid side chain, a segment of the structure, or the protein as a whole (33). To achieve the best results and get the target protein structure as close to the real structure as possible, it was necessary to optimize the protein structure as a whole using energy minimization. All structures were optimized in a periodic boundary water box using the Gromacs 4.6 force field, with a Steepest Decent optimization of 5,000 steps followed by a conjugate gradient optimization of 2,000 steps. This parameter configuration ensures that the protein structure was completely optimized. Unreasonable dihedral angles, side-chain structures, and excessively close contacts are eliminated during optimization, resulting in a more rational protein structure.

Tricarboxylate citrate transporter templates cannot be used directly for multimer modeling predictions due to their poor homology. As a result, molecular docking was used to predict the Tct multimeric structure model. The multimers are all dimeric, according to the template protein structure. As a result, molecular docking predicted a direct binding model of the two Tct subunits to construct dimers. The dimeric Tct protein structure was also optimized using the Gromacs 4.6 kinetics software, yielding the optimized Tct dimeric protein 3D structure.

### Molecular Docking and Molecular Dynamics Simulations

Tricarboxylate citrate transporter protein was set as the receptor and the citric acid and malic acid small molecules as the ligands in AutodockTools (34). The binding modes of citric acid and malic acid at the Tct active site were searched for using molecular docking calculations, and the lowest energy binding mode was chosen for visualization with the PyMol program to provide insight into the binding interactions of different ligands with proteins during the transport process.

Simulation of kinetics: Citric acid (CIT) and malic acid (MAL) small molecule three-dimensional structure files were created using Chemdraw and combined with molecular docking results, and the energy minimum conformation of the protein-citric acid complex was calculated and output for molecular dynamics simulations. Gromacs 2018 created the initial model structure for the transmembrane molecular dynamics simulation; first, the cell membrane-protein-small molecule composite system was built based on the energy minimum complex conformation obtained through docking, and the cell membrane was a phospholipid bilayer of DOPC and DOPG (7:3). The cell membrane-transport protein-small molecule complex model was then subjected to a 30 ns molecular dynamics simulation in an aqueous environment system using a Gromacs-based solvent model. The force field Gromos 53A6 was used, and the water model was SPC. The simulated system employs a standard cubic box that is wrapped around the model and other molecules, with the complex in the center of the box. Before subjecting the model to a completely free kinetic simulation, the complex was optimized for 2,000 steps using the steepest descent method to eliminate possible atomic collisions. Following that, the protein was positioned and molecular dynamics simulations for the solvent were performed for 100 picoseconds (ps); then the protein backbone and ligand were restricted for 100 ps; finally, the restriction was removed and the simulation was performed for 100 ps, i.e., the pre-procedure. The simulated system's long-range van der Waals forces were set to 1.4 nm, and the classical interactions were calculated using the spherical “cut-off” radius method. The simulations were run in steps of 2 fs, with one conformation output every 100 ps, using periodic boundary conditions in all directions. Gromacs 2018 was used to trace the simulations, and PyMol and vmd were used to visualize them. The root mean square deviation (RMSD) can approximate the system's relative change in conformation and is an important criterion for determining whether the simulated system converges. As a result, RMSD is used in this research work to determine and judge the system's equilibrium moment.

### Statistical Analysis

All statistical data from three independent values were analyzed by one-way analysis of the variance (ANOVA) with multiple comparison tests (Tukey's) using SPSS 16.0.

The data is presented as mean standard deviation (SD). The differences were statistically significant at *P* < 0.05.

## Results

### Experimental Validation of Tct

Our previous research on mitochondrial transporters revealed the presence of two MCTs (Tct and Ct) in oleaginous *M. circinelloides* WJ11, which are associated with high lipid accumulation (15, 17). The molecular investigation of citrate efflux from the mitochondria by Tct in *M. circinelloides* was first investigated in this study. For the mechanistic study of Tct, and the citrate transporter gene (*tct* of WJ11) was expressed and purified in *E. coli*. The protein's citrate transport activity was investigated in Tct reconstituted liposomes.

**Figure 1:**
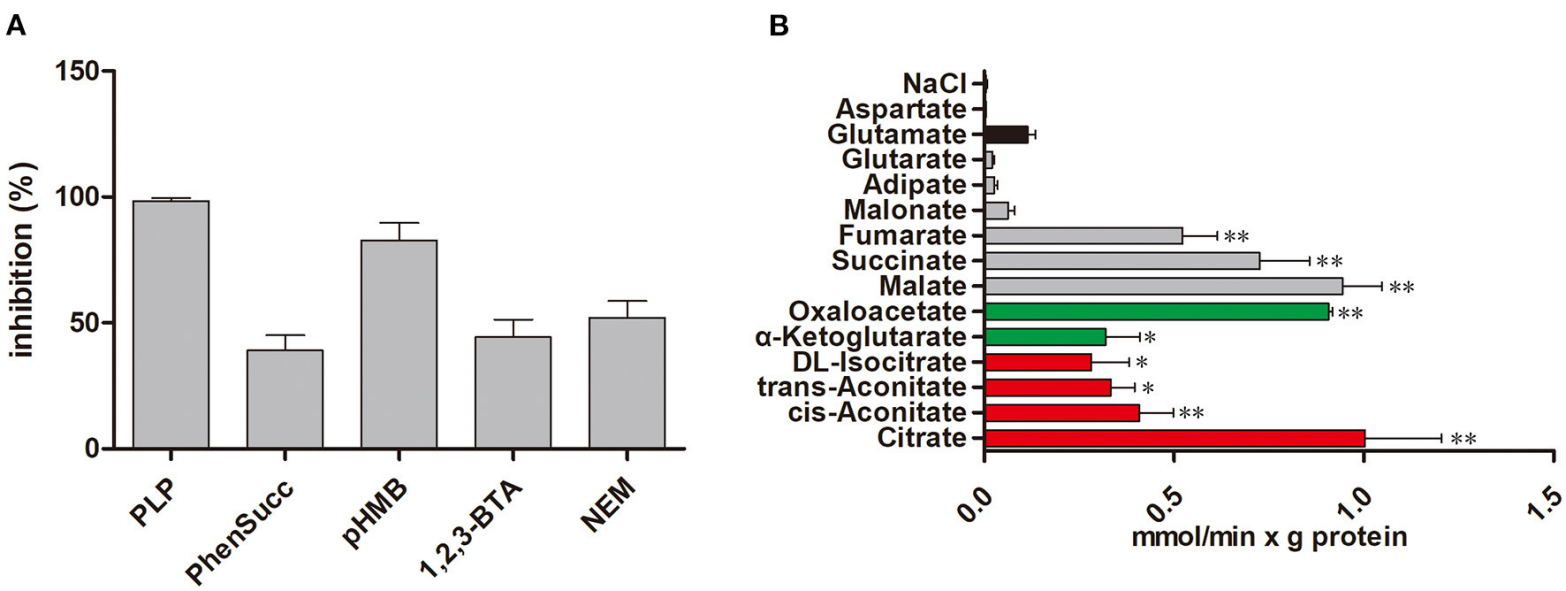
Transport properties of Tct. **(A)** Effect on citrate/citrate exchange of Tct by inhibitors. Liposomes were reconstituted with Tct and preloaded internally with 10 mM citrate. Transport was initiated by the addition of 0.01 mM [^14^C] citrate and terminated after 2 min. The concentrations of the inhibitors were 20 mM (PLP, pyridoxal 5′-phosphate), 2 mM (phesucc, phenylsuccinate), 0.1 mM (pHMB, p-hydroxymercuribenzoate), 2 mM (1,2,3-BTA, 1,2,3-benzenetricarboxylate), 1 mM (NEM, N-ethylmaleimide) **(B)** Tct transport activity is substrate dependent. Tct-reconstructed liposomes were preloaded internally with 20 mM substrates (Red: tricarboxylic acids. Green color: α-ketodicarboxylic acids. Gray color: dicarboxylic acids. Black color: other compounds). Transport was initiated by the addition of external 0.01 mM [^14^C] citrate. The values are means ± SEM of three independent experiments. *represents significant difference *p* < 0.05 while as **represents *p* < 0.01.

**Figure 2:**
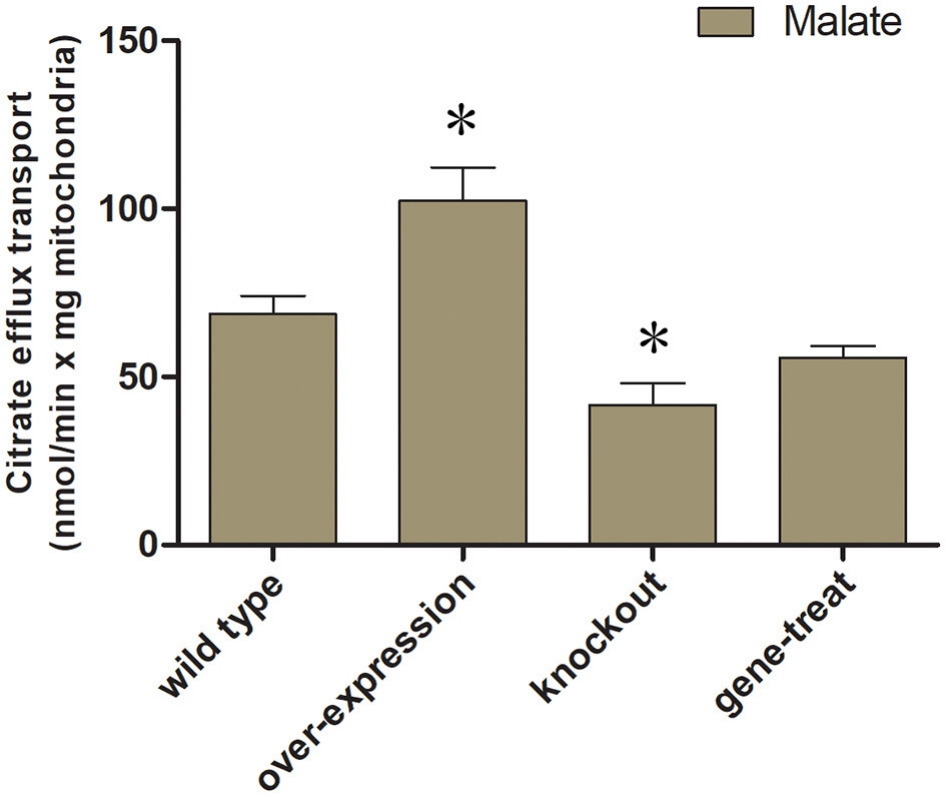
Mitochondrial transport activity of transformed *M. circinelloides* strains. Mitochondria from the wild-type transformants, overexpressed and knockout were preloaded with 0.01 mM [14C] citrate. For exchange malate were added as substrates (10 mM malate) outside of mitochondria. Error bars represent standard deviations (*n* = 3). *represents significant difference *p* < 0.05.

We examine the impact of certain MCT inhibitors on the recombinant Tct-catalyzed [^14^C] citrate/citrate exchange reaction (Figure 1A). Pyridoxal-5'-phosphate (PLP) and pHMB strongly inhibit Tct transport activity, that is why PLP was utilized as a reaction termination inhibitor. The homo-exchange activity of Tct at internal and external concentrations of 10 μM and 10 mM of [^14^C] citrate and citrate, respectively, were inhibited by PLP. The highest absorption activity of [^14^C] citrate in proteoliposomes was achieved by internal citrate, malate, oxaloacetate, succinate, and fumarate. [^14^C] citrate is also exchanged, to a less extent, with internal α-ketoglutarate, isocitrate, and aconitate (Figure 1B). Our results showed that Tct has a high efficiency value for [^14^C] citrate/citrate exchange with Km 0.018 mM at 25 °C. Furthermore, the *tct* overexpression and knockout plasmids were created and transformed into *M. circinelloides* WJ11. A modified K & R medium with glucose as the sole carbon source was used to grow these engineered strains. The mitochondria of transformed strain were isolated, and their transport activity was investigated. The current study used ^14^C metabolic flux analysis on recombinant *M. circinelloides* strains to examine the effects of *tct* gene overexpression or knockout on metabolic fluxes using calculated extracellular flux values. In the presence of 10 mM malate, the mitochondria of the *tct*-overexpressing transformant showed a 49% increase in [^14^C] citrate efflux, whereas the mitochondria of the *tct*-knockout transformant showed a 39% decrease in citrate efflux compared to the mitochondria of wild-type WJ11 (Figure 2). These findings support the importance of Tct in citrate efflux from the oleaginous fungus *M. circinelloides*, which is associated with high lipid accumulation.

### Tct Sequence Characteristics

**Figure 3:**
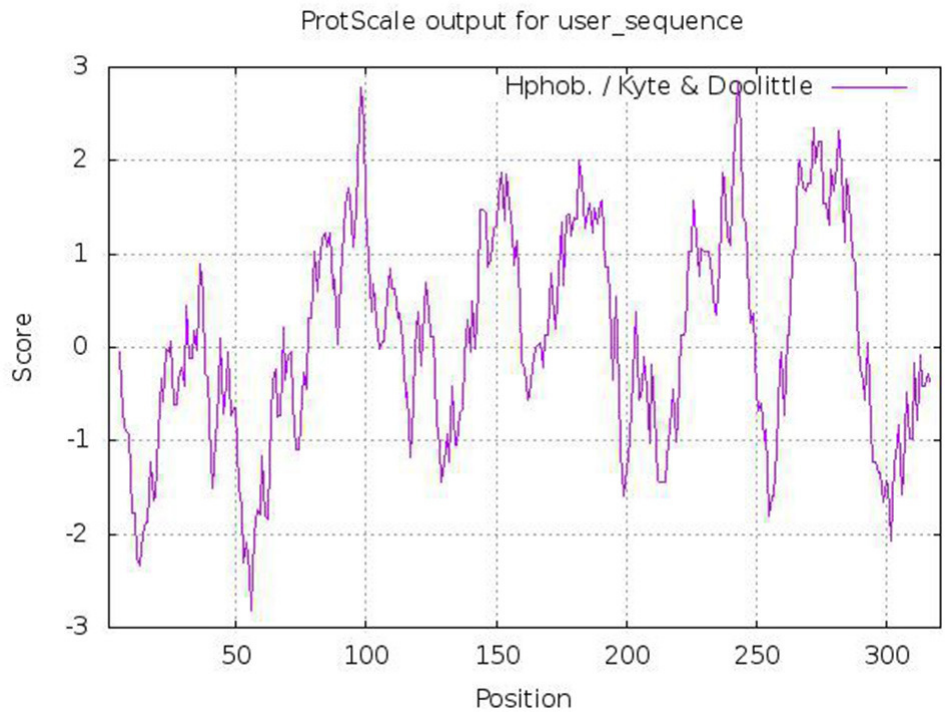
Predicted hydrophobicity analysis of Tct protein heavy chains (>0 indicates hydrophobicity; <0 indicates hydrophilicity).

The *tct* genes of WJ11 encode protein with a total of 321 amino acid residues. The sequence similarities between Tct and Ct proteins were found to be 39.6%. The Tct protein was found to contain 12.1% of positively charged amino acid residues (Arg+His+Lys) and 7.8% of negatively charged amino acid residues (Asp+Glu). The hydrophobic residues in Tct protein were high ≈50.6% which provided a foundation for the stable embedding of transmembrane proteins into non-polar lipid membrane (Figure 3). The significant hydrophobicity (total average hydrophilic value of 0.089) of Tct protein provides a basis for embedding in the cell membrane and functioning as a stable transporter protein in biological cells.

### Homology Modeling Analysis

Models were created by manually aligning Tct protein sequence of WJ11 to each of the available templates. Although the protein sequence comparison with the other template sequences in NCBI revealed low homology that prevents reliable alignment. As a result, cycles of computerized modeling followed by manual alignment improvement were performed until a model that satisfies data and constraints was generated. Based on the 10 selected Protein Data Bank (PDB) structures, the 3D structure prediction of Tct was performed. The template sequences all covered the entire Tct sequence provides a basis for structure prediction by the threading method (fold recognition), which improves the accuracy of the predicted structures. Because of the low sequence homology models based solely on sequence alignment were not expected to produce good results. Manual alignment optimization avoided this issue to some extent and enabled the generation of an acceptable model for Tct protein.

**Figure 4:**
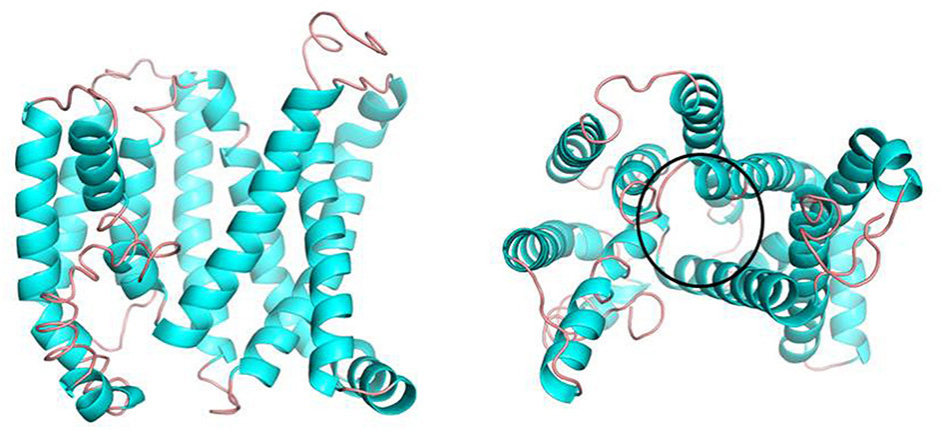
Side view (left) and top view (right) of the 3D structure of the Tct model.

Tricarboxylate citrate transporter is a transmembrane protein that facilitates the transport of molecules therefore its transmembrane structure prediction is of great significance. The results of the transmembrane structure prediction of Tct by TMHMM (http://www.cbs.dtu.dk/services/TMHMM) are shown in Figure 4. Tricarboxylate citrate transporter contains three transmembrane structures (i.e., 141–163, 178–195, and 262–284), while the chain structures 75–125 and 225–250 also have a 50% probability of being transmembrane structures. The overall structure of the protein is V-shaped and intra-membrane.

**Figure 5:**
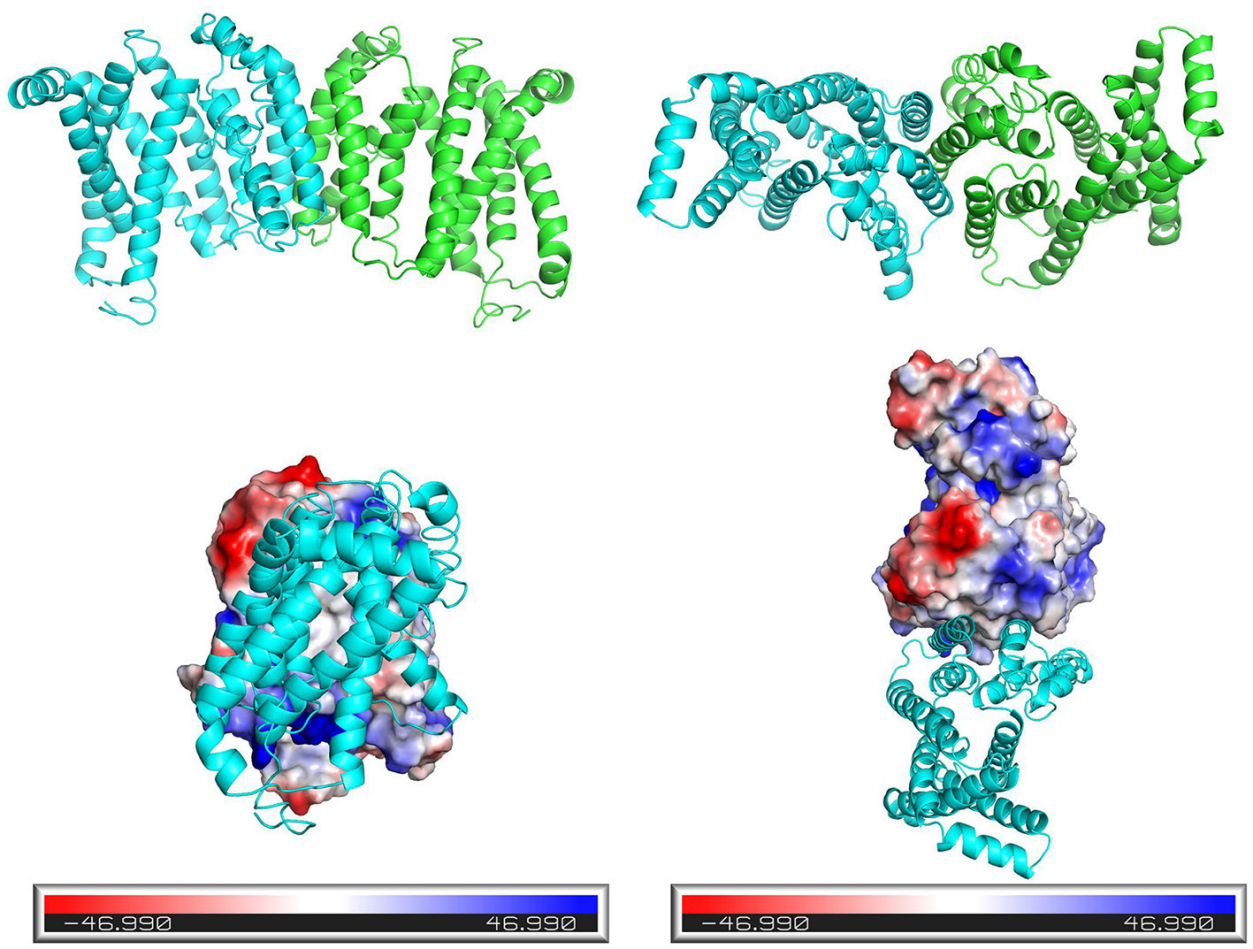
Side view (left) and top view (right) of the three-dimensional structure of the dimer formed by the two subunits of Tct, and electrostatic surface view of the monomeric protein.

Figure 5 depicts the three-dimensional structure of the Tct dimeric protein. Tricarboxylate citrate transporter's two subunits dimerize in a spatially complementary manner, with the lateral helical chains inserted into each other's subunit vacancies, forming good spatial complementarity. The electrostatic surface of the Tct monomer, with the positively charged region in blue, the negatively charged region in red, and the white region (large portion) is the non-charged or very low charge region, i.e., the non-polar region; thus, the Tct surface is densely packed with hydrophobic residues. The two subunits aggregate via hydrophobic interactions on the subunit surface, promoting multimer aggregation and binding stability. Figure 5 also depicts the binding electrostatic surface of Tct's two subunits. Tricarboxylate citrate transporter's binding interface is only in the non-polar hydrophobic region, according to the electrostatic analysis of the binding interface.

### 3D Structure Prediction and Active Site Analysis

**Figure 6:**
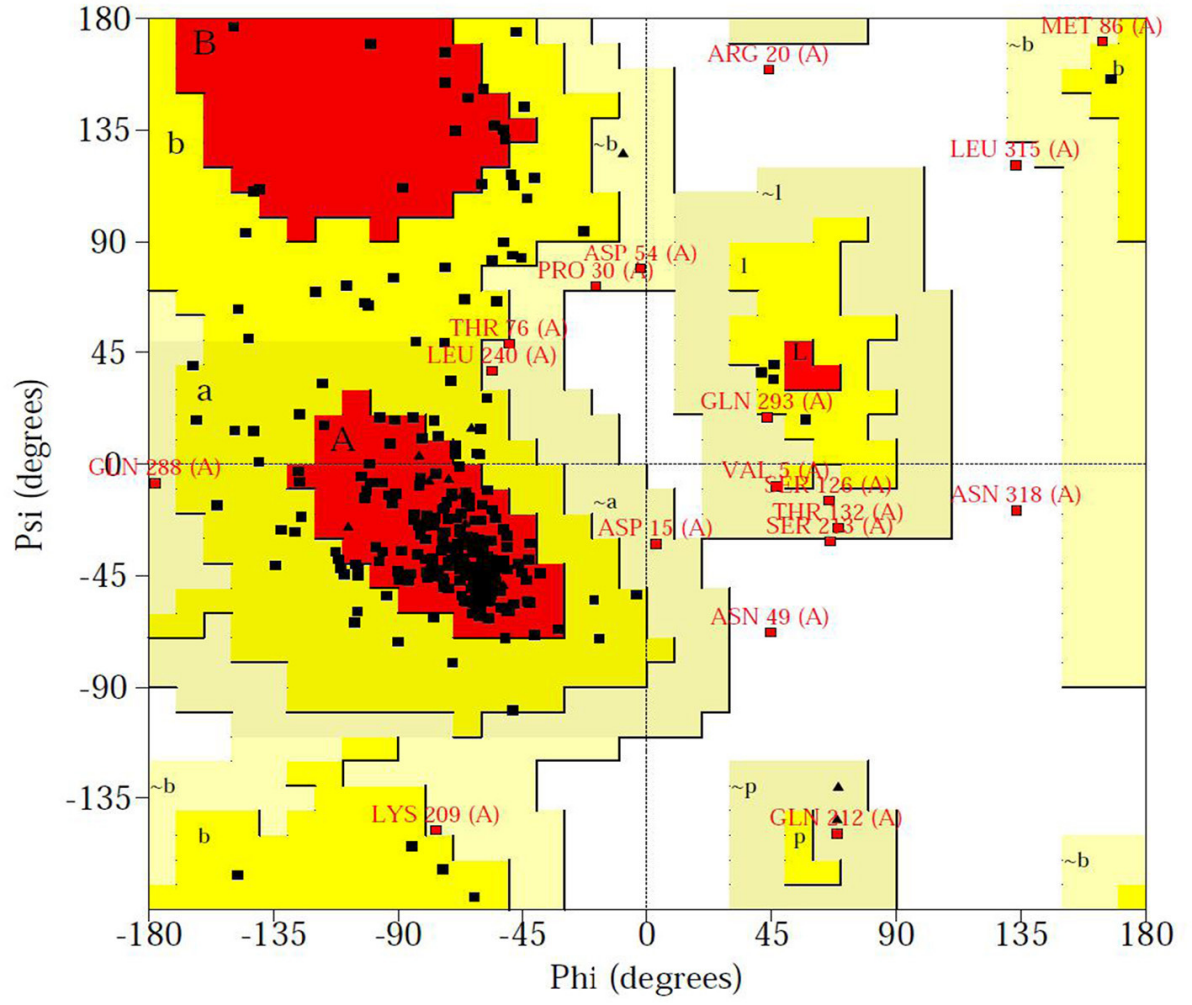
Ramachandran plot of Tct protein structure obtained from Procheck program.

The Ramachandran plot of amino acid residues obtained from PROCHECK program evaluated the constructed Tct protein structure as shown in Figure 6. The model Tct structure contains 70.7% of the amino acids in the core region, 23.3% of the amino acid residues in the allowed region, 4.2% of the amino acid residues in the maximum allowed region, and only 1.8% of the amino acid residues in the forbidden region of the torsion angle. Although the Tct whole protein chain segment model contains five residues in the forbidden region of the torsion angle, this is due to the greater flexibility of this region of the protein. Because the protein as a whole follows the stereochemical energy rules, the constructed protein model has a reasonably accurate stereological structure.

**Figure 7:**
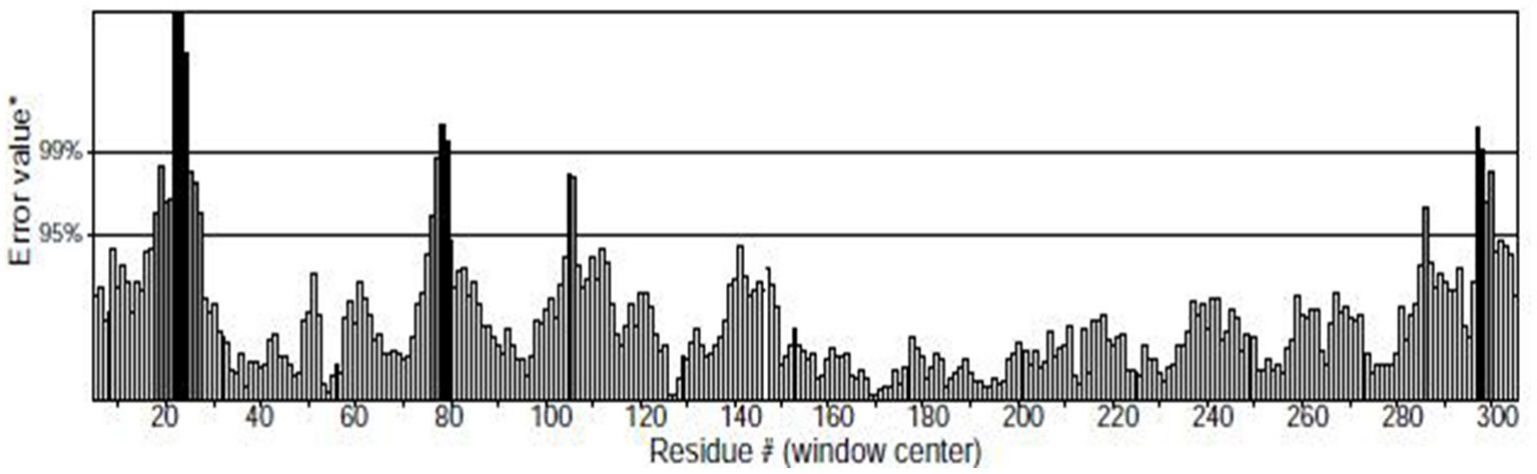
ERRAT diagram of the Tct protein model.

The higher the Overall quality factor value, the better the result, generally up to one for high resolution crystal structures and only around 91% for average resolution. Figure 7 shows the results of the ERRAT analysis for the Tct protein, with an ERRAT value of 91.374 indicating the high accuracy of the model generated by the structure prediction. The two error limits present in the graph indicate how likely it is that the region above the line is problematic, and below the error, line indicates a protein structure with high resolution. Based on this result, it can be seen that the ERRAT values for amino acid residues near residues 21–28 in the Tct protein chain segment are above the two error lines due to the high flexibility of this region due to the Loop; the rest of the structures are below the error lines.

### Molecular Docking With Citrate and Malate

Using Autodock's molecular docking, the interaction of citric acid and malic acid with Tct transporter proteins was investigated at the molecular level. Tricarboxylate citrate transporter had binding energy of −4.87 kcal/mol to citric acid, while −3.80 kcal/mol to malic acid. The lower the binding energy, the more stable is the binding of a molecule to the protein. Citric acid was found more stable binding than malic acid.

**Figure 8:**
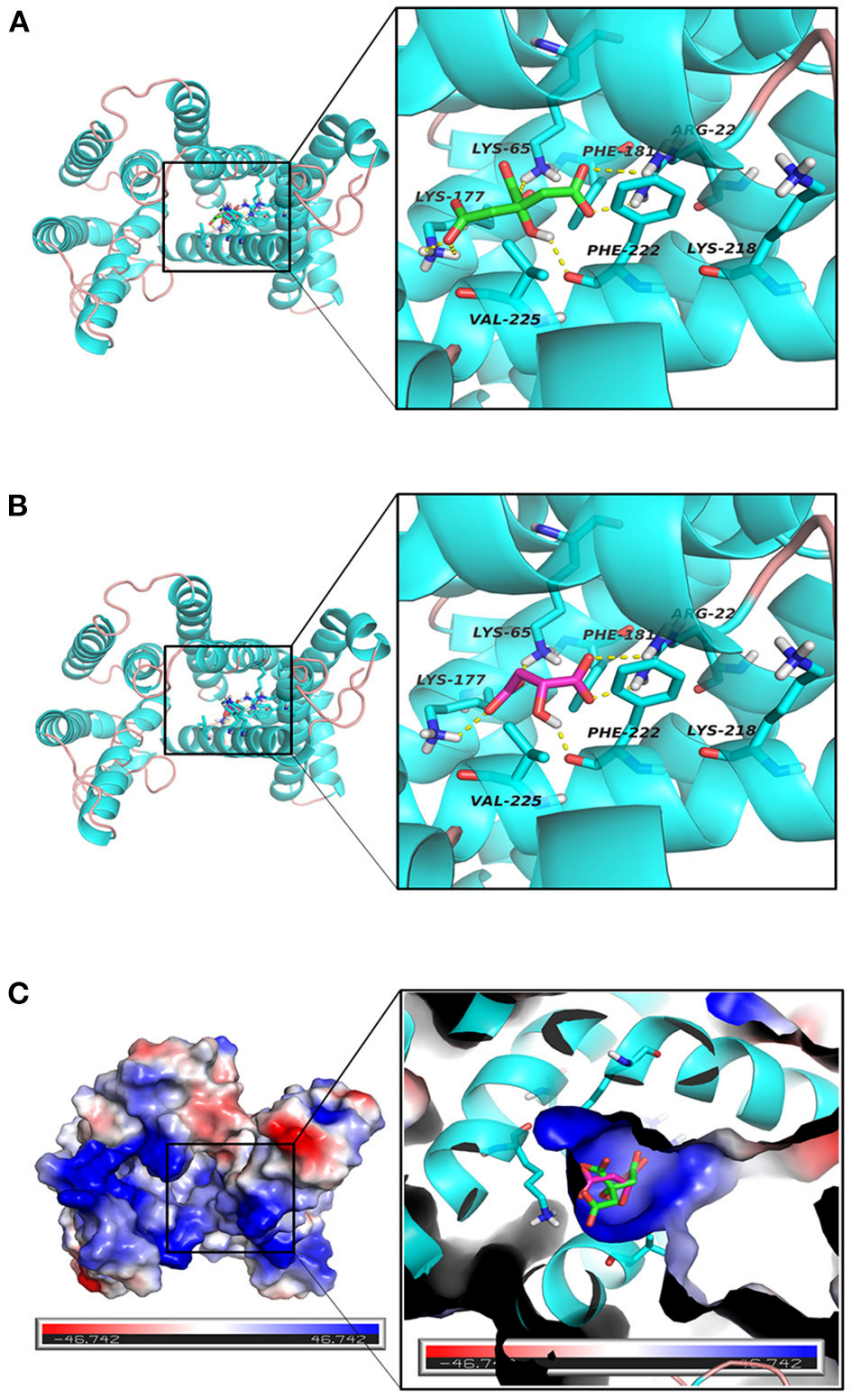
**(A–C)** Tct Transport Protein (Cyan), citrate (green), malate (red) binding interaction diagram and three-dimensional structure diagram of the active site.

Figure 8 show the binding patterns of citric and malic acids in the Tct protein active site. As can be seen from Figure 8, citric and malic acids can be stably bound in the Tct transporter protein active pocket by interaction with residues after deprotonation. Comparative analysis revealed that citric acid can interact with key residues ARG22, LYS65, LYS177, and PHE222 in the Tct active site by hydrogen bonding, with double hydrogen-bonding interactions with ARG22 and LYS177, allowing citric acid to be stably bound at the bottom of the active pocket, providing for its transport. In contrast, malic acid can also hydrogen-bond with ARG22, LYS65, LYS177, and the key residue PHE222 in its binding in the Tct active site, but only the double hydrogen-bonding interaction with LYS177 is present, resulting in a slightly less stable binding than that of citric acid. This is one of the reasons why malic acid has a higher docking binding energy than citric acid.

The electrostatic matching of citric and malic acids with Tct is depicted in Figure 8. The electrostatic surface distribution map of Tct shows that the majority of the interior of the Tct transporter protein's active cavity is a positively charged region, which provides the electrostatic environment for stable binding of citric and malic acids after deprotonation. As a result of the deprotonation of the citric and malic acid carboxyl groups, the polycarboxyl functional groups are negatively charged and can bind strongly electrostatically to the positive region at the active site's bottom. Because citric acid contains three deprotonated carboxyl groups, the electrostatic binding interaction with Tct proteins is stronger, improving binding stability and lowering binding energy, providing a theoretical explanation for the small molecule transport mechanism and transport efficiency.

### Molecular Dynamics Simulation of Tct Protein Interacting Small Molecules

**Figure 9:**
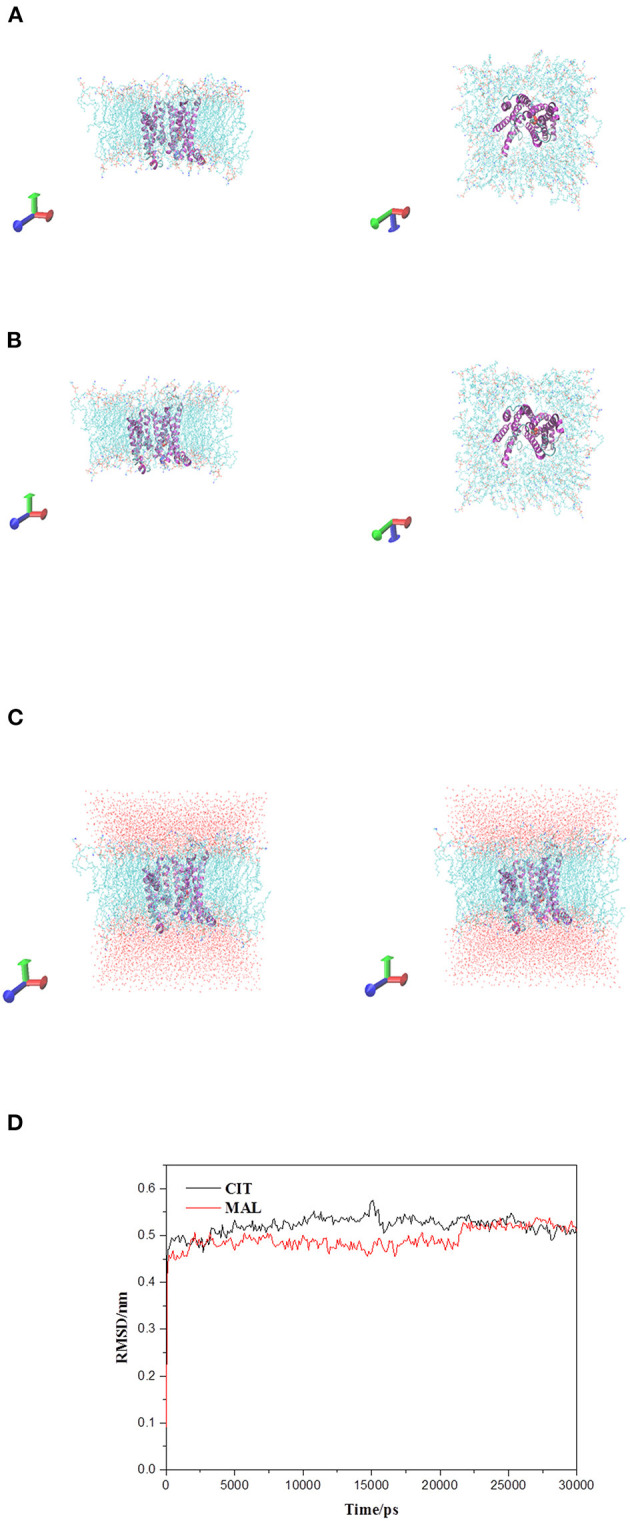
Tct protrin and substrates complex and membrane system. **(A)** Tct-CIT was complex and membrane system. **(B)** Tct-MAL was complex and membrane system. **(C)** The solvation model of Tct-CIT (left) and Tct-MAL (right) system. **(D)** RMSD of backbone vs. simulation time. Side view left and top view right for **(A)** and **(B)**.

**Figure 10:**
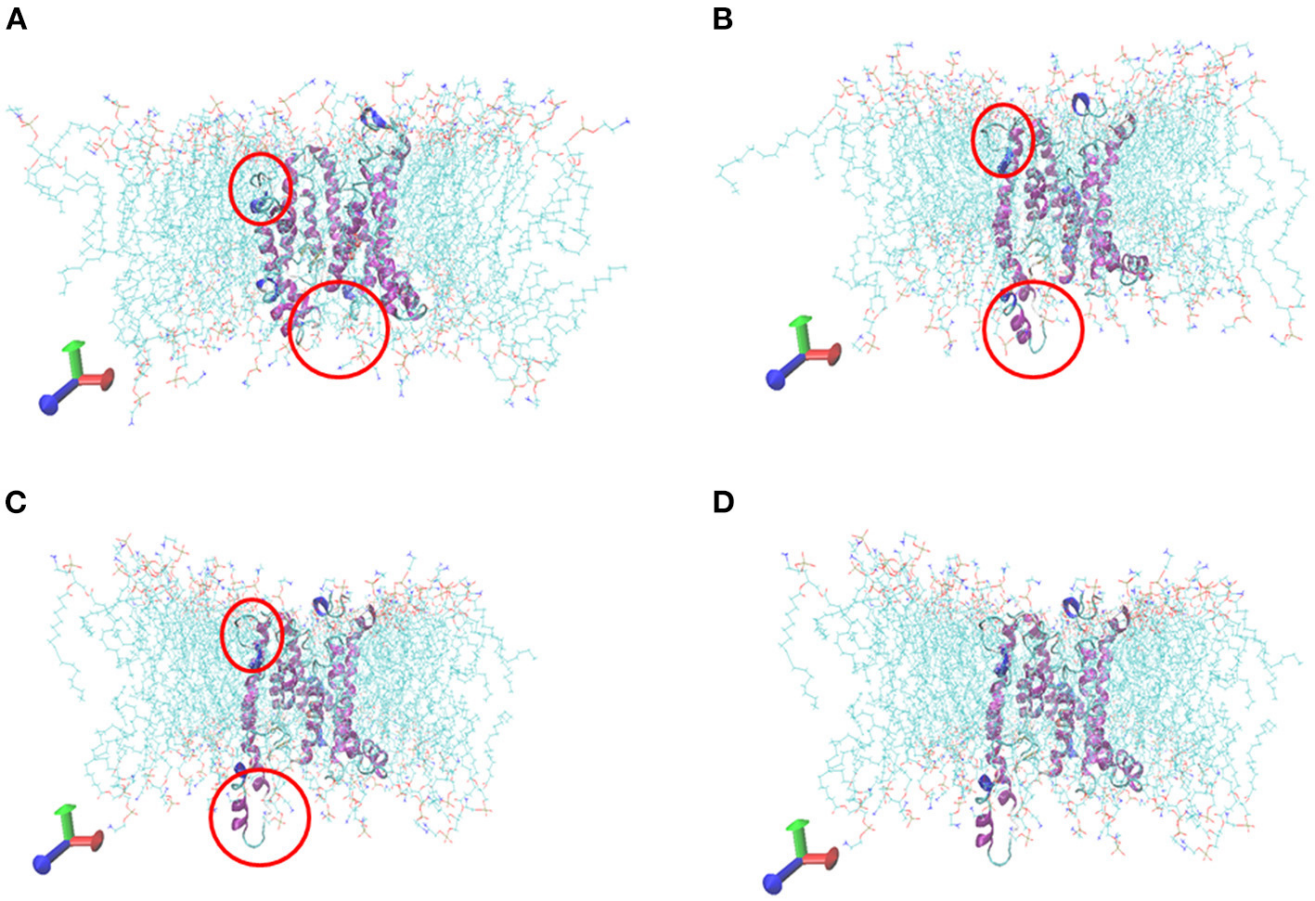
Comformations of Tct-CIT system vs. MD simulation time. **(A)** 0 ns, **(B)** 10 ns, **(C)** 20 ns, **(D)** 30 ns, Tct was in cartoon, membrane in line, ligand in ball.

**Figure 11:**
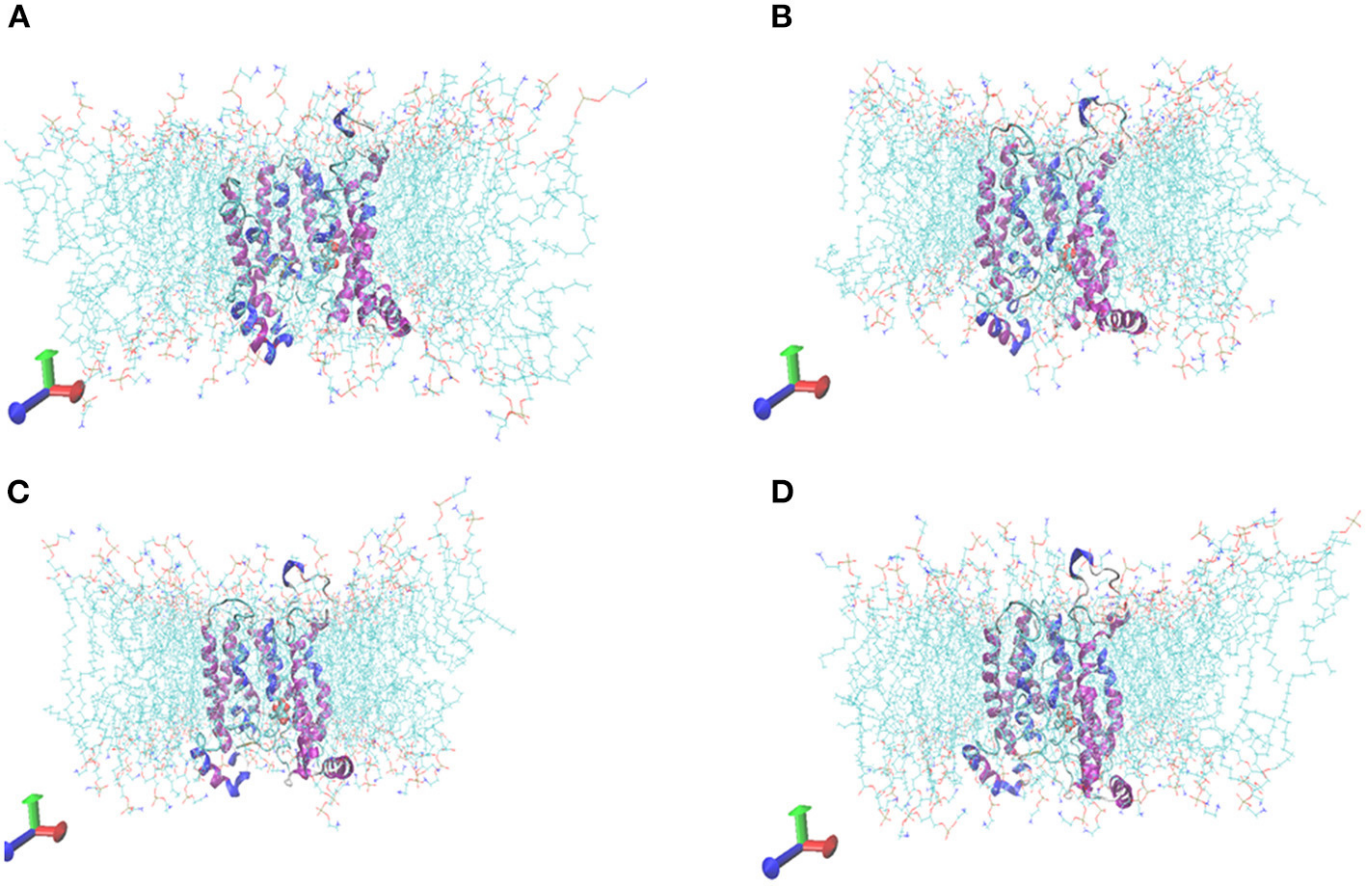
Comformations of Tct-MAL system vs. MD simulation time. **(A)** 0 ns, **(B)** 10 ns, **(C)** 20 ns, **(D)** 30 ns, Tct was in cartoon, membrane in line, ligand in ball.

**Figure 12:**
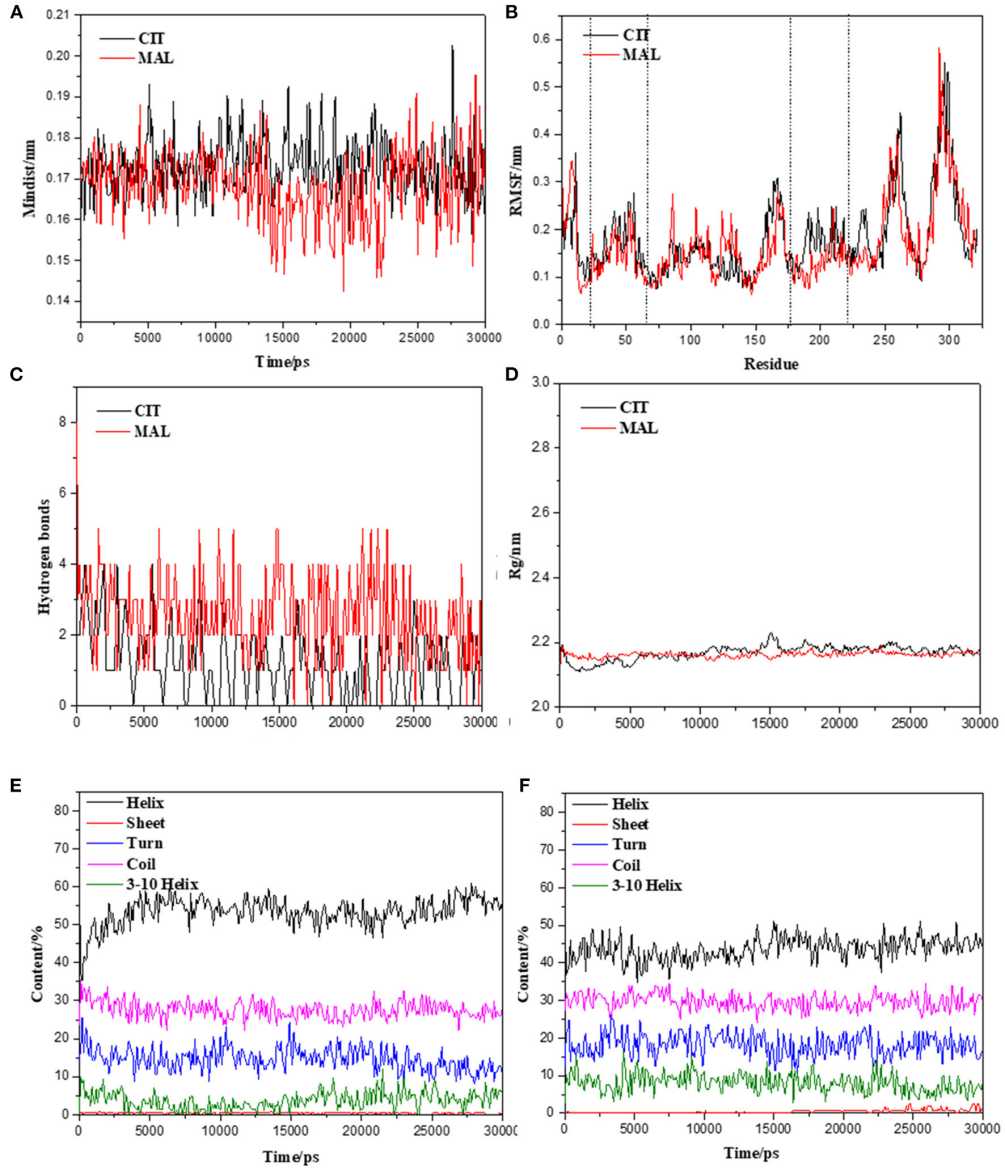
Molecular dynamics simulation stability assessment of Tct protein interacting small molecules complex system. **(A)** Min-distance of skeletal muscle myosins multimer vs. simulation time of Tct-CIT and Tct-MAL. **(B)** RMSF of protein in the complex system vs. simulation time. **(C)** Intramolecular hydrogen bonds between protein and ligand vs. simulation time. **(D)** Radius of gyration of skeletal muscle myosins multimer vs. simulation time. Secondary structure content of Tct during the simulation with and without electric field vs. simulation time, **(E)** for citrate and **(F)** for malate.

The initial RMSD of the complexes fluctuated considerably due to the protein-small molecule cell membrane interactions, the adjustment and adaptation of the systems to the solvent effect; after 10 ns, both the Tct-CIT and Tct-MAL systems reached equilibrium and the RMSD of the systems stabilized at around 0.4–0.5 nm, fluctuating within 0.1 nm. The molecular dynamics simulations in the solvent systems were all stable. The kinetic simulations for the Tct-CIT and Tct-MAL systems were carried out to extract the conformational structures for a more detailed comparative analysis, and the results are shown in Figures 9–11. As can be seen from Figure 10 the Tct-CIT system showed some degree of fluctuation in protein structure during the kinetic process due to the effect of cell membrane and solubilization. During the kinetic simulation, the helical chain of Tct underwent helicalization, followed by unhelicalization and post-helicalization (Figure 10). However, the overall structure of Tct did not change much and was relatively stable, while the Tct-MAL system was relatively stable during the kinetic simulations, with no significant changes in the overall tertiary structure of the protein (Figure 11). At the same time, the binding of CIT and MAL, respectively, to Tct remained relatively stable during the kinetic simulations, and there was no dissociation. To further determine the binding of CIT and MAL to Tct, the minimum distances between the small molecules and Tct during the kinetic simulations were analyzed, and it was found that the minimum distances between the small molecules and the protein were both stable at around 0.17 nm (Figure 12), with little difference. The distance is just one hydrogen bond distance, i.e., the two are more stable in the transport protein and there is no dissociation.

The radius of gyration trajectories of the proteins can indicate the stability of the proteins during the kinetic process, and also the degree of protein tightness. As can be seen from Figure 12B, the radius of gyration of the translocated proteins in the Tct-CIT and Tct-MAL systems was essentially constant throughout the kinetic simulations under the interaction of solubilization and cell membranes; the Rg of the same protein was also essentially constant in the simulations calculated for different systems. The Rg of Tct in the Tct-CIT and Tct-MAL systems was also relatively small after equilibration, at around 2.2 nm, and fluctuations can also be small. Thus, solubilization and cell membrane interactions did not lead to any adjustment of the protein structure and did not affect the transport process of small molecules, thus allowing efficient transport of small molecules with good activity.

Figure 12 shows the secondary structure content of Tct in the Tct-CIT and Tct-MAL systems during the kinetic simulations; in the Tct-CIT system, there was a significant increase in the Helix content of Tct, with significant fluctuations (Figure 12E), due to the unfolding of the Sheet content into helical chains, while the random loop Coil content was largely unchanged. In contrast, in the Tct-MAL system, the Sheet chain unfolded to a lesser extent, resulting in a less pronounced increase in Helix, while the other chain segments remained essentially unchanged (Figure 12F). Thus, although there is some change in the secondary structure of Tct in both systems, there is also no increase in flexibility and the overall structural stability of the protein is relatively good, resulting in high transport efficiency.

## Discussion

Oleaginous microorganisms have high lipid content and are thought to be potential cell factories for the production of high-value FAs (35, 36). These microbes have become industrial targets for lipid products and are amenable to metabolic engineering to increase their FA content (37, 38). Various studies proved that FA synthesis is triggered by citrate accumulation in the mitochondria, the relationships between lipid production and the activities of related enzymes in the TCA cycle and glycolysis have been extensively studied (39–41). Furthermore, the mitochondrial citrate transport system is expected to play a significant role in lipid production by controlling the citrate between mitochondria and the cytoplasm. As a result, it was necessary to investigate the mitochondrial citrate transport system that will be the key targets for metabolic engineering that in turn will help to understand the mechanism of lipid accumulation in oleaginous microorganisms. However, only a few reports on mitochondrial Cts in microbes have been published, and little is known about the mechanism of the mitochondrial citrate transport system in oleaginous microorganisms (42). *Mucor circinelloides* WJ11 that accumulates higher levels of lipids has been used as a model organism to study citrate transport system. In our on-going research on mitochondrial transporters, we identified 51 transporter genes that were predicted to be involved in a variety of important metabolic pathways, including oxidative phosphorylation, the citric acid cycle, FA oxidation, and amino acid degradation (15). WJ11, a high lipid-producing strain of *M. circinelloides*, was shown to have higher citrate flux from the mitochondria to the cytoplasm than CBS 277.49, a low lipid-producing strain (43). In WJ11, five genes were identified coding for transporters of the mitochondrial citrate transport system (15). In previous research, we found that CT of WJ11 contributes to the efflux of citrate from mitochondria and supply enough carbon sources for cell utilization in normal physiological processes and lipid biosynthesis (17). In the present work, we targeted *tct* gene that was overexpressed and its roles in FA accumulation and related metabolic pathways under nitrogen limitation were investigated. In WJ11, *tct* was successfully overexpressed and their FA content was increased while as knock out of *tct* resulted in slight decrease of lipid production. These findings supported the hypothesis that CTP overexpression increased lipid accumulation in *M. circinelloides* (16). Lipid accumulation is efficient when citrate is present in the cytosol of oleaginous microorganisms (39). The efflux of citrate from the mitochondria to the cytoplasm is a key event during lipogenesis in oleaginous microorganisms (5, 6). In theory, overexpression of citrate carriers promotes citrate transport from the mitochondria to the cytoplasm, thereby hastening the formation of the cytoplasmic citrate pool (44).

In the present study, the transporter Tct of *M. circinelloides* WJ11 has been cloned, overexpressed purified, kinetically and structurally characterized. The Tct protein was expressed in *E. coli*, isolated, and functionally reconstituted in a liposomal system. The Km-value of Tct for the citrate was found to be 0.018 mM. Based on Km-values, our findings showed that Tct's affinity to citrate was significantly higher than as previously reported in *S. cerevisiae* Yhm2p for citrate and oxoglutarate were about 0.16 and 1.2 mM, respectively (42).

The mitochondria of the *tct*-overexpressing transformant of *M. circinelloides* WJ11 showed a 49% increase in citrate efflux, whereas the mitochondria of the *tct*-knockout transformant showed a 39% decrease in citrate efflux compared to the mitochondria of wild-type WJ11. Our results are in confirmation with previous research that showed overexpression of MT in *M. circinelloides* resulted in increased citrate efflux from mitochondria (43). A higher citrate pool serves as an acetyl-CoA donor, promoting FA synthesis. The molecular mechanism by which the mitochondrial citrate transport system increases citrate efflux from the mitochondria and its role in FA synthesis is still under investigation.

The lack of an experimentally determined structure is one of the difficulties in studying Tct. To develop tool compounds in structure-based ligand discovery, a thorough understanding of the substrate binding site is required. The use bioinformatics in Tct are sufficient to identify the intrinsic role of the Tct in *M. circinelloides* that demonstrated a strategy for selecting candidate genes for further functional investigation. This will allow for further research and development of future Tct. As a result, we modeled Tct with new alignment and template structures and investigated their potential utility in small molecule discovery. A 3D model of the yeast mitochondrial Tct protein was also constructed using homology modeling. The WJ11 genes *tct* encode proteins with a total of 321 amino acid residues that contains 12.1% of positively charged amino acid residues and 7.8% of negatively charged amino acid residues. Most mitochondrial carrier family proteins (MCF) are small, with a length of around 300 amino acids and a molecular weight ranging from 30 to 35 kDa (45). The hydrophobic residues in Tct protein were high ≈50.6% with an average hydrophilic value of 0.089. The transmembrane structure prediction of Tct contains three transmembrane structures (i.e., 141–163, 178–195, and 262–284), while the chain structures 75–125 and 225–250 also have a 50% probability of being transmembrane structures. The overall structure of the protein was found V-shaped and its 3D structure is dimeric. It is now thought that MCs exist and function as monomers. The only carrier that has been proven to exist as a homodimer is the human aspartate-glutamate carrier (with two isoforms: AGC1 and AGC2) (46, 47). When eel liver mitochondria were solubilized with the mild detergent digitonin, the dimeric form of the Tct protein was discovered (48). Molecular docking and molecular dynamics were also used to investigate molecular interactions and the mode of binding between Tct and citrate. Tricarboxylate citrate transporter had binding energy of −4.87 kcal/mol to citric acid vs. −3.80 kcal/mol to malic acid. The molecular dynamics analyses revealed that the complexes had certain conformational stability, which was due to the significant interactions between Tct and citrate, which were consistent with the docking study interactions.

## Conclusion

MCTs are so important in cellular bioenergetics, our group has been studying their structure-based mechanism. Tricarboxylate citrate transporter overexpression boosted citrate transport from the mitochondria to cytosol, which is one of the reasons of high lipid accumulation in the oleaginous fungus *M. circinelloides* WJ11. Tricarboxylate citrate transporter appears to play a key role in citrate transport in *M. circinelloides*, based on kinetics data collected in this study in conjunction with our homology-modeled Tct structure. The findings of this study show the significance of the *tct* gene as a target gene for genetic engineering to improve citrate transport in oleaginous fungus, hence opening up new perspectives for improving *M. circinelloides* WJ11 for commercial lipid production.

## Data Availability Statement

The original contributions presented in the study are included in the article/Supplementary Material, further inquiries can be directed to the corresponding author/s.

## Author Contributions

WY and AS planned the experiments, carried out the experimental work, and generated the figures. SD, CS, and HZ did additional experimental work and participated in writing of the article. HM, XG, and HF participated in the writing of the article. YS supervised the work and participated in the writing of the article. All authors contributed to the article and approved the submitted version.

## Funding

This work was supported by National Science Foundation of China (grant nos. 31972851 and 31670064), TaiShan Industrial Experts Programme (tscy no. 20160101), Shandong Provincial Key Technology R&D Plan (nos. 2018GNC110039 and 2018GSF121013).

## Conflict of Interest

The authors declare that the research was conducted in the absence of any commercial or financial relationships that could be construed as a potential conflict of interest.

## Publisher's Note

All claims expressed in this article are solely those of the authors and do not necessarily represent those of their affiliated organizations, or those of the publisher, the editors and the reviewers. Any product that may be evaluated in this article, or claim that may be made by its manufacturer, is not guaranteed or endorsed by the publisher.
