# Supplementary material for: Tricarboxylate Citrate Transporter of an Oleaginous Fungus Mucor circinelloides WJ11: From Function to Structure and Role in Lipid Production
Source: Front Nutr. 2021 Dec 9;8:802231. doi: 10.3389/fnut.2021.802231 (PMC8696028; doi:10.3389/fnut.2021.802231)

Table S1: Modeling calculations of Tct

| Protein | PDB Hit | Iden1 | Iden2 | Cov | Norm.Z-score |
| --- | --- | --- | --- | --- | --- |
| TCT | 6ezuA | 0.13 | 0.19 | 0.89 | 0.63 |
|  | 3mktA | 0.1 | 0.23 | 0.86 | 0.98 |
|  | 6r9tA | 0.07 | 0.4 | 0.97 | 0.75 |
|  | 5bu0A | 0.13 | 0.14 | 0.84 | 0.6 |
|  | 2k0m | 0.15 | 0.07 | 0.14 | 0.75 |
|  | 6oh2A | 0.11 | 0.19 | 0.84 | 0.76 |
|  | 2pff | 0.16 | 0.33 | 0.97 | 1.37 |
|  | 4y5jA | 0.14 | 0.18 | 0.62 | 0.53 |
|  | 2pff | 0.18 | 0.33 | 0.85 | 0.82 |
|  | 1lshA | 0.21 | 0.21 | 0.88 | 0.93 |

Figure S1: whole cell lysate of Tct over-expression in *Ecoli.* analyzed by SDS-PAGE and Western blot

PC_1_ PC_2_ M_1_ NC 1 2 NC_1_ NC_2_ 3 4 5 6

kDa

120

80

60

40

30

20

10

M_2_ NC 1 2 3 4 5 6

kDa

120

80

60

50

42

32

18


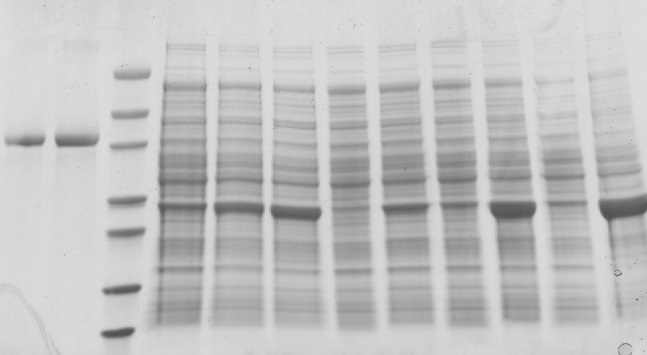

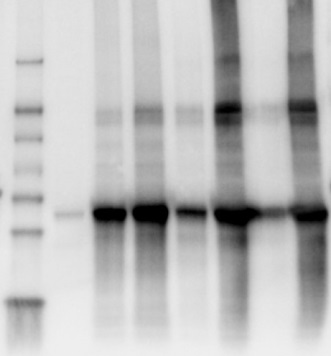


Figure S2: SDS-PAGE & Western blot Analysis of purified Tct


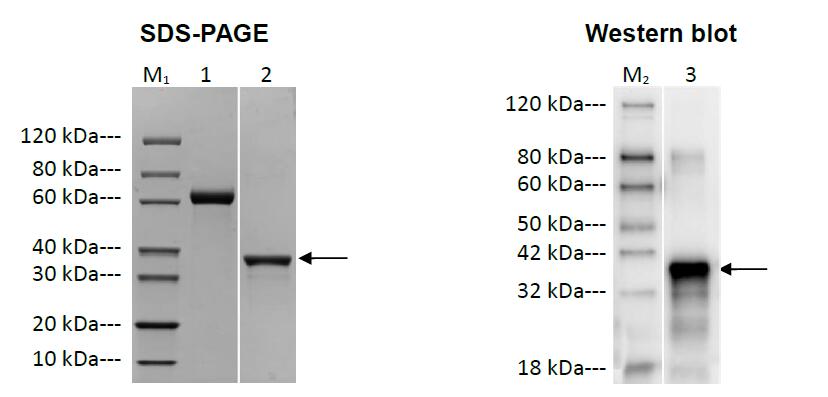

Supplement: Supplementary file 1 [file Data_Sheet_1.docx]
